# GafChromic XR‐QA film in testing panoramic dental radiography

**DOI:** 10.1120/jacmp.v8i2.2457

**Published:** 2007-04-19

**Authors:** Robert Y.L. Chu, Tin Lam, Yongguang Liang

**Affiliations:** ^1^ Department of Radiological Sciences University of Oklahoma Health Science Center, Veterans Affairs Medical Center Oklahoma City Oklahoma U.S.A.; ^2^ University of Oklahoma Health Science Center Radiology Service, Veterans Affairs Medical Center Oklahoma City Oklahoma U.S.A.

**Keywords:** panoramic radiology, radiochromic film, dose–area product, DAP

## Abstract

The location and field size of the incident X‐ray beam in panoramic dental radiography cannot be ascertained visually most of the time. However, these parameters are needed for quality control and dosimetry determination. To alleviate this problem, we tested GafChromic XR‐QA film on two panoramic systems. For each system, we used the length of a cross‐sectional image of the incident beam and the exposure measurement with a pencil ion chamber to compute the dose–area product. The result was confirmed by direct analysis of a dose distribution on a film. Placement of the ion chamber was determined by the latter images. The GafChromic XR‐QA version of radiochromic film has thus been demonstrated to usefully complement a pencil ion chamber in the testing of a panoramic radiography system.

PACS numbers: 87.52.Df, 87.59Bh, 87.66.Cd, 87.66Jj

## I. INTRODUCTION

Panoramic radiography in dentistry poses several challenges for medical physics testing. The X‐ray tube revolves around a patient's jaw while continuously emitting radiation through a slit. The physicist may not be able to hold the X‐ray tube stationary for an evaluation of beam quality or a determination of radiation dosimetry. Goldstein^(1)^ therefore developed a jig to help with some of the quality control measurements. However, he also demonstrated the inaccuracies of thimble ion chambers in measuring radiation exposure.^(2)^

That deficiency has now perhaps been overcome by the more recent proposal of Perisinakis and colleagues^(3)^ to use a pencil ionization chamber, which has a more uniform response along the length of the sensitive volume. Those authors demonstrated the use of such a detector to determine dose–area product (DAP) and the subsequent computation of effective dose from the DAP. The necessary measurements, plus an alignment of the X‐ray beam, require knowledge of the location of the aperture in the X‐ray tube assembly and the beam entrance at the imaging detector; however, the aperture is not necessarily visible. With the move to digital technology, film processors, and hence dental films or general diagnostic radiography films, will no longer be available to help with these tests.

A new type of radiochromic film (GafChromic XR‐QA dosimetry film: International Specialty Products, Wayne, NJ) with better sensitivity at low radiation was introduced quite recently. In the present investigation, we evaluated the use of this new film to record the location and dimensions of the X‐ray beam in a medical physics evaluation.

## II. MATERIALS AND METHODS

We used two panoramic systems in this investigation. System A (Planmeca Promax: Planmeca Oy, Helsinki, Finland) is a newer system. Neither the beam‐exit opening in the X‐ray tube assembly nor the collimator in front of the film cassette holder is visible. System B (GX1000 Model 46: Gendex Dental Systems, Lake Zurich, IL) is an older system. A collimator is clearly seen in front of its film‐holding drum.

We created a step wedge–like reference pattern as described in an earlier publication.^(4)^ Pieces of GafChromic XR‐QA film that constituted the pattern were exposed to various known levels of radiation from a Philips Optimus X‐ray system (Philips Medical Systems, Andover, MA) at 81 kV photons (kVp). This reference pattern and the images acquired later for DAP computation were scanned on a flatbed scanner (ScanMaker 9800 XL: Microtek USA, Carson, CA) in RGB mode at a resolution of 100 pixels per inch. The red components of the images, being more sensitive to radiation^(^ ^4^ ^,^ ^5^ ^)^, were used for analysis. We used fourth‐order polynomials to fit pixel values of the reference steps to the corresponding known levels of radiation exposure. With that calibration, the images obtained as described below were converted to maps of dose distribution in air kerma.^(6)^

**Figure 1:**
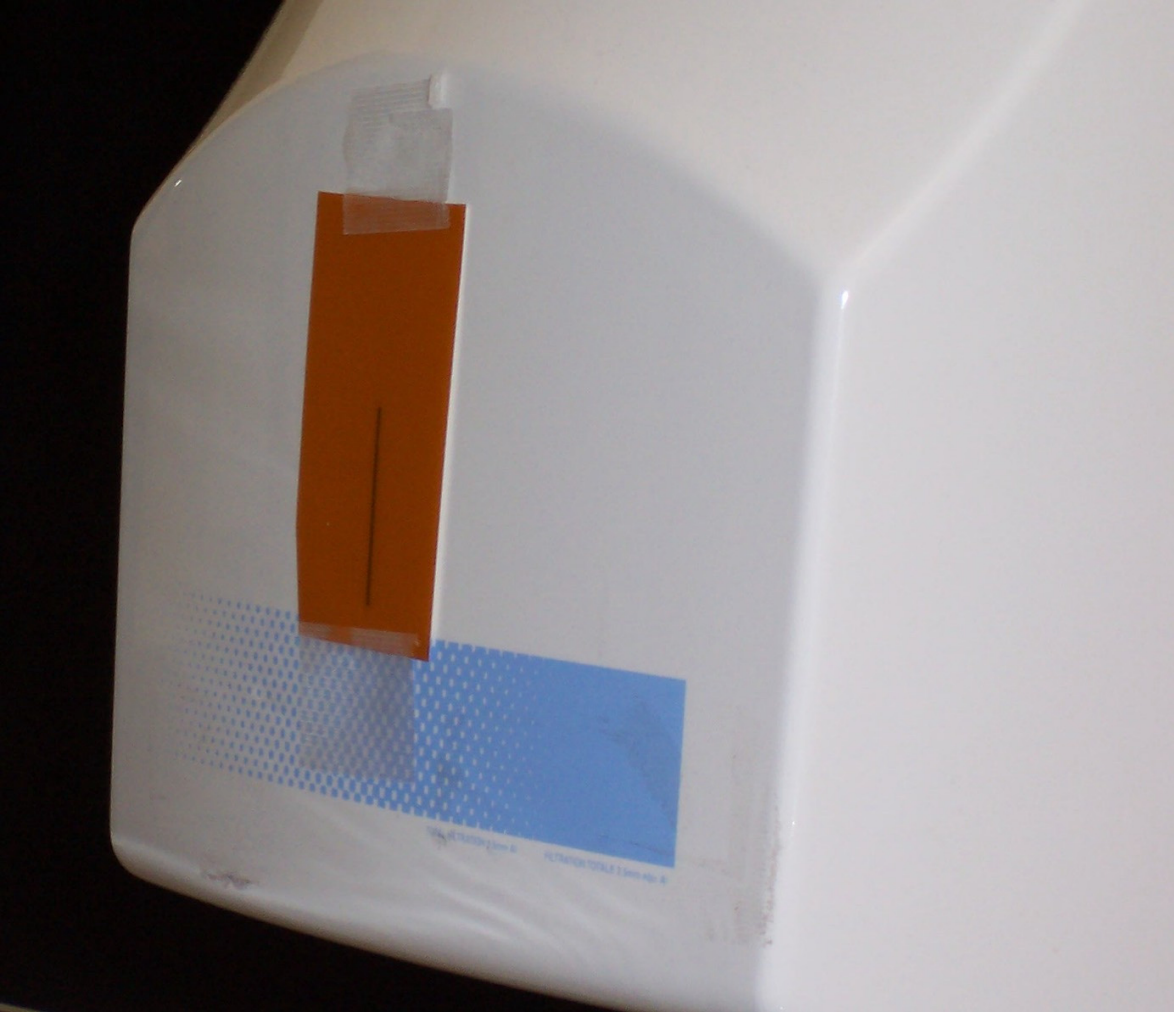
Film placed on the cover of the X‐ray tube assembly. A dark image of the pre‐patient collimator was visible after irradiation.

In the discussion that follows, we call the slit‐like aperture at the X‐ray tube assembly a “pre‐patient collimator” and the aperture to the imaging detector (film screen or otherwise), a “post‐patient collimator.” A 5.0×7.5‐cm piece of film was placed over the likely area of the pre‐patient collimator (Fig. 1). Immediately after one panoramic scan without any object in the path of the X‐ray, an image of the collimator was clearly visible. The exposure technique was 68 kVp, 7 mA, and 16 s for system A and 0 kVp, 4 mA, and 18 s for system B. Then, we placed a pencil ion chamber (model 10X5–3CT: Radcal Corporation, Monrovia, CA), connected to a Radcal radiation monitor (model 1515), centrally over and horizontally to the mid‐length of the previously exposed image. The panoramic scan was repeated, and the exposure was recorded.

**Figure 2:**
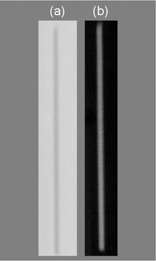
Image acquired on radiochromic film in front of the post‐patient collimator. (a) Red component of the image presented in grayscale. (b) Converted to dose (in air kerma) distribution (grayscale was adjusted for presentation).

A 5×15‐cm piece of film was placed on the likely position of post‐patient collimator. Three scans were performed to create one image of sufficient visibility on this film. The radiochromic film was then scanned 5 days post irradiation. Using a previously described method,^(6)^ we converted the red component of the image [Fig. 2(a)] to dose distribution in air kerma [Fig. 2(b)]. The DAP was computed from the latter value and then divided by three to obtain the value for one panoramic scan.

## III. RESULTS

Dose–width product (DWP) was computed using the pencil ion chamber reading and the length of the chamber's sensitive volume.^(3)^ Multiplying DWP by the length of the image of the pre‐patient collimator, we obtained the DAP at the pre‐patient collimator. This value, which is supposed to be invariant with respect to distance from the radiation source, was compared to the DAP computed from film at the post‐patient collimator. The results showed agreement within experimental uncertainties (Table 1).

**Table 1:** Comparison of computing dose–area product (DAP) with pencil ion chamber and GafChromic XR‐QA dosimetry film (International Specialty Products, Wayne, NJ)

				$\text{DAP}\;(\text{cGy}•{\text{mm}}^{2})$ using	
Panoramic system	kVp	mA	Time (s)	Pencil ion chamber	GafChromic film
A	68	7	16	713	697
B	90	4	18	721	763

kVp=kilovolt−peak.

## IV. DISCUSSION

Following the prescription of Perisinakis et al.,^(3)^ we used the lengths of the radiation field at the pencil ion chamber to compute DAP. When the physical apertures are recessed from the nearest approachable surfaces of the given X‐ray tube assembly, these lengths can be significantly different from the specifications provided for pre‐patient collimators. The improvement in our method is the use of radiochromic film, which provides instant results, instead of dosimetric film, which requires wet processing. The radiochromic film thus helps with proper placement of the ion chamber.

In the present experiment, the time between irradiation and scanning of the film was determined by the availability of the computer–scanner system. Images should be stable during these few days.^(4)^ The uncertainty in the measurement of the length of the pre‐patient collimator could be a fraction of a millimeter. Thus the error introduced to DAP measurement by the pencil ion chamber would be compatible with that of DAP measurement using film.^(6)^ However, the latter is a much more complicated process; it was performed in the present investigation solely for the purpose of verifying the validity of the former method.

Goldstein, in his proposal on quality control testing,^(1)^ used a fluorescent screen to align the ion chamber. The use of radiochromic film to record the position of the X‐ray beam may be a more convenient alternative. In our evaluation of radiochromic film, we placed a piece of film behind the post‐patient collimator (not shown) of system B and successfully demonstrated a misalignment of the X‐ray beam with respect to the post‐patient collimator.

## V. CONCLUSIONS

We demonstrated the usefulness of radiochromic film as a complement to a pencil ion chamber in the testing of panoramic radiography machines. Imaging at the pre‐patient collimator with this type of film is a simple way to obtain the length of the collimator for a computation of DAP with a pencil ion chamber. The resulting image also shows the location of the collimator and therefore helps in the proper placement of the ion chamber for exposure measurement. A piece of radiochromic film can also be placed at the post‐patient collimator to test the alignment of the X‐ray beam.

## ACKNOWLEDGMENT

Radiochromic film used in this investigation was provided by David Lewis, PhD, of International Specialty Products.
